# Optical Coherence Tomography and Visual Evoked Potentials as Prognostic and Monitoring Tools in Progressive Multiple Sclerosis

**DOI:** 10.3389/fnins.2021.692599

**Published:** 2021-08-05

**Authors:** Simone Guerrieri, Giancarlo Comi, Letizia Leocani

**Affiliations:** ^1^Experimental Neurophysiology Unit, San Raffaele Hospital, Institute of Experimental Neurology (INSPE), Milan, Italy; ^2^Vita-Salute San Raffaele University, Milan, Italy; ^3^Casa di Cura del Policlinico, Milan, Italy

**Keywords:** multiple sclerosis, progressive multiple sclerosis, visual pathway, visual evoked potentials, optical coherence tomography

## Abstract

Understanding the mechanisms underlying progression and developing new treatments for progressive multiple sclerosis (PMS) are among the major challenges in the field of central nervous system (CNS) demyelinating diseases. Over the last 10 years, also because of some technological advances, the visual pathways have emerged as a useful platform to study the processes of demyelination/remyelination and their relationship with axonal degeneration/protection. The wider availability and technological advances in optical coherence tomography (OCT) have allowed to add information on structural neuroretinal changes, in addition to functional information provided by visual evoked potentials (VEPs). The present review will address the role of the visual pathway as a platform to assess functional and structural damage in MS, focusing in particular on the role of VEPs and OCT, alone or in combination, in the prognosis and monitoring of PMS.

## Background

### The Challenge of Progressive Multiple Sclerosis

Multiple sclerosis (MS) is a chronic inflammatory, immune-mediated disease of the central nervous system (CNS; Ontaneda and Fox, 2015), characterized by demyelination, axonal loss, and neurodegeneration. Although the pathophysiology underlying the different phenotypes still needs to be clarified, four main clinical courses of the disease have been identified: relapsing–remitting MS (RRMS; characterized by clearly defined neurological exacerbations with full or incomplete recovery, in the presence of dissemination in space and time of the inflammatory process among the CNS), clinically isolated syndrome (CIS; a first neurological episode suggestive of MS, but formal criteria of dissemination in time are not fulfilled), secondary progressive MS (SPMS; defined retrospectively by the occurrence of gradual disability worsening with or without occasional relapses, minor remissions, and plateaus, following an initial RRMS course), and primary progressive MS (PPMS; characterized by progressive accumulation of disability from disease onset with occasional plateaus, temporary minor improvements, or acute relapses still consistent with the definition; Lublin and Reingold, 1996; Lublin et al., 2014). The MS courses can be further qualified by the presence/absence of disease activity [presence of relapses and/or magnetic resonance imaging (MRI) activity – i.e., gadolinium-enhancing lesions or new/unequivocally enlarging T2 hyperintense lesions] and by the disability state: worsening, improving, or stable (Lublin et al., 2014).

The pathological key features underlying the clinical expression of the disease can be depicted as a spectrum, ranging from waves of acute focal inflammation in RRMS to predominant neurodegenerative features with concomitant chronic compartmentalized inflammation in progressive multiple sclerosis (PMS) (Lassmann et al., 2007; Giovannoni et al., 2016).

During the past decades, a major progress has been made in understanding disease mechanisms in RRMS, with inflammation and subsequent focal demyelination with breakdown of the blood–brain barrier representing the main driver of clinical disease in this subset of patients. This knowledge has led to the development of anti-inflammatory and immunomodulatory treatments that effectively reduce the severity and frequency of new demyelinating episodes (Diebold and Derfuss, 2016). In PMS, instead, focal disruption of the blood–brain barrier is less common, and widespread degeneration of the white and gray matter variably combined with slow expansion of chronically active lesions are the pathological hallmarks (Lassmann, 2017). Several and non-necessarily exclusive mechanisms have been proposed to explain the pathogenesis of PMS (i.e., compartmentalized ongoing chronic inflammation, chronic inflammation leading to inflammation-independent neurodegeneration, and primary neurodegeneration amplified by concurrent independent inflammation), with SPMS and PPMS course likely sharing similar pathophysiological features (Confavreux and Vukusic, 2006; Trapp and Nave, 2008; Frischer et al., 2009; Lassmann et al., 2012). Fundamental pathogenetic pathways responsible of clinical progression, however, still remain unidentified, with no available accurate preclinical model reproducing this stage of the disease. The approval of Ocrelizumab for active PPMS and SPMS treatment (Montalban et al., 2017), and of Siponimod for active SPMS by EMA and for relapsing MS by FDA (Kappos et al., 2018), represent important encouraging novelties, but the tangible real-world impact of these molecules has still to be assessed especially in the absence of overt inflammation (Montalban et al., 2017; Kappos et al., 2018). Unfortunately, previous studies exploring neuroprotective strategies have failed; however, some positive results have recently emerged from phase III clinical trials and are now under exploration in definite clinical trials (Ontaneda et al., 2015; Sorensen et al., 2020). Moreover, the process of discovery of new therapeutic targets for PMS is a priority of the International Progressive MS Alliance (2021), a multistakeholder initiative promoted by International Federation of Multiple Sclerosis and MS patient associations.^1^

### The Visual Pathway as a Model of Brain Damage in Multiple Sclerosis

In order to succeed in the challenge represented by PMS, our ability to early detect the pathological processes on the stage will be of fundamental importance. At present, diagnosis of PMS is mainly retrospective since imaging methods as well as other biomarkers to catch or predict progression are not well established (Correale et al., 2017). There is an unmet need for new strategies to identify inflammation/demyelination and particularly neurodegeneration in a subclinical phase, with consequent prompt interventions aimed to prevent disability to occur for our patient.

Emerging evidence suggests that the visual system may play an important role in this game (Martinez-Lapiscina et al., 2014). The visual pathway is in fact frequently involved in MS, with visual dysfunction that is not only common but also highly relevant (Fisher et al., 2006; Heesen et al., 2008; Chatziralli et al., 2012). Furthermore, the visual pathway may represent a model of both acute focal CNS damage [through acute optic neuritis (ON) and retinal periphlebitis] (Albrecht et al., 2007; Siger et al., 2008), as well as a model of chronic, diffuse CNS involvement (through chronic retinopathy, optic neuropathy, and *trans*-synaptic degeneration). The ongoing pathological processes can be accurately evaluated due to the availability of highly sensitive imaging [i.e., MRI or optical coherence tomography (OCT)] and electrophysiological [i.e., visual evoked potentials (VEPs) and electroretinography (ERG)] tests. The combination of these techniques allows to describe the interactions between the different processes at play (such as inflammation, demyelination, and axonal and neuronal loss) *in vivo* and in a non-invasive way, features that identify the visual pathway as an elective platform to differentiate MS pathophysiology from other inflammatory conditions of the CNS (Vabanesi et al., 2019), as well as a reliable model to monitor the disease and to test new neuroprotective or regenerative therapies in the context of clinical trials (Fisher et al., 2006; Heesen et al., 2008; Chatziralli et al., 2012; Martinez-Lapiscina et al., 2014; Villoslada, 2016).

Optical coherence tomography in MS has been widely used to measure in particular retinal nerve fiber layer (RNFL) and ganglion cell–inner plexiform layer (GCIPL) thickness as markers of neuroaxonal loss, allowing to detect subclinical neurodegeneration (Petzold et al., 2010; Alonso et al., 2018; Costello and Burton, 2018). RNFL and GCIPL thickness have been correlated with tests of visual function (Pulicken et al., 2007; Pueyo et al., 2008; Zaveri et al., 2008), with global disability scores such as Expanded Disability Status Scale (EDSS; Albrecht et al., 2007; Siger et al., 2008), with functional measures as those provided by VEPs (Klistorner et al., 2008; Pueyo et al., 2008; Di Maggio et al., 2014), but also with cerebral and optic nerve MRI parameters (Trip et al., 2006; Grazioli et al., 2008; Siger et al., 2008), as well as with fluid biomarkers such as serum neurofilament light chain concentration (Tavazzi et al., 2020). Most of the evidence available in the field is actually related to the RRMS course, with neuroretinal atrophy being associated with disease activity (Pisa et al., 2017), but with the possibility to detect RNFL and GCIPL thinning over time in MS patients with progression independent of relapse activity (PIRA; Bsteh et al., 2020; Pisa et al., 2020). Cross-sectional RNFL, total macular volume (TMV), and GCIPL thickness measures independently predicted long-term disability in large cohorts of predominately RRMS patients (Martinez-Lapiscina et al., 2016; Rothman et al., 2019; Lambe et al., 2021), while the application of mathematical models has suggested RNFL evolution, resulting from a mix of inflammatory and degenerative processes, to accurately reflect disability progression over time (Montolío et al., 2019).

More recently, other retinal layers have also received attention as possible biomarkers in MS: in particular, inner nuclear layer (INL) consists of a network of bipolar, amacrine, and horizontal cells; despite some signs of atrophy have been described on histopathology at this level in MS (Green et al., 2010), *in vivo* studies did not show an extensive INL atrophy as in the case of RNFL and GCIPL, even after ON (Seigo et al., 2012; Syc et al., 2012). Pathology studies have identified inflammation and microglial activation within the inner retina in MS patients (Green et al., 2010), and *in vivo* observations also suggest INL as a possible biomarker of inflammation within the CNS, with increased INL thickness reflecting a condition of retinal inflammation, which parallels brain inflammatory activity in MS: microcystic macular edema (MME) within this layer has in fact been described to be associated with ON and disability; furthermore, increased INL thickness has been associated with a greater risk of developing new T2 or gadolinium-enhancing lesions and of new relapses (Saidha et al., 2012; Balk et al., 2019); finally, successful response to disease-modifying treatments (DMTs) has been associated with a sustained reduction of INL volume (Knier et al., 2016). Other authors, however, have postulated the possibility for INL thickening (and MME in particular) to be related to vitreomacular tractions, Müller cell pathology, subclinical uveitis, or retinal periphlebitis, conditions possibly found in association with MS (Kerrison et al., 1994; Chen and Gordon, 2005). Significant correlations between INL thickening and RNFL/GCIPL thinning have been also described: according to this evidence, it has been speculated that INL enlargement is related to structural changes in other retinal layers (as the result of the opposing tractions between inner limiting and Bruch’s membranes), being therefore compensatory in nature (Kaushik et al., 2013).

MRI can be used to identify inflammation (lesion load on T2-weighted images and gadolinium-enhancing lesions on T1-contrast sequences), but also (with 3D high-resolution T1-weighted images) to quantify regional atrophy along the visual pathway, such as optic nerve atrophy after ON, of the lateral geniculate nucleus (LGN) at the thalamic level and of the visual cortex (Gabilondo et al., 2014). Other MRI parameters such as the magnetization transfer ratio (MTR) and the diffusion-weighted imaging (DWI) are sensitive to microstructural damage, allowing to characterize demyelination and axonal damage along the visual pathway, with an association with visual function measures (Melzi et al., 2007; Naismith et al., 2010; Kolbe et al., 2012). In the following sections, possible relations between OCT-VEPs parameters and MRI data have been assessed; however, an extensive dissertation of MRI findings and their implications in PMS is beyond the purpose of the present review.

Among functional techniques, traditional full-field VEPs (ff-VEPs) can be performed as an indicator of demyelination/remyelination, expressed by latency delay/shortening of the major component P100, with potential diagnostic, prognostic, and monitoring roles in MS (Comi et al., 1999; Leocani et al., 2018). In addition, multifocal techniques (mf-VEPs) allow to assess conduction for separate portions of the visual field, providing information about local signals of small areas occupying up to 24 central degrees of the visual field, with the possibility to detect mild abnormal local responses and scotomas (Klistorner et al., 2008).

Starting from this background, in this article, we wanted to assess the real value of the visual pathway as a specific biomarker of functional and structural damage in PMS patients, focusing in particular on VEPs and OCT use as possible prognostic and monitoring tools.

## Evidence Acquisition

We searched PubMed up to March 15, 2021, using the following terms: “Progressive Multiple Sclerosis and Visual Evoked Potentials,” “Progressive Multiple Sclerosis and Optical Coherence Tomography,” “Optical Coherence Tomography and Disability and Multiple Sclerosis,” and “Visual Evoked Potentials and Disability and Multiple Sclerosis.”

## Visual Evoked Potentials in PMS

There is little specific information about VEPs in PMS, and especially PPMS, because many studies on VEPs in MS were performed prior to the current classification of disease courses (Lublin et al., 2014).

Currently available data on ff-VEPs sensitivity mainly derive from studies assessing the role of a multimodal neurophysiological assessment in MS cohorts, including subsets of PMS patients. Leocani et al. (2006) performed a study in which, among the others, 41 PMS patients (13 PPMS and 28 SPMS) underwent multimodal evoked potentials including ff-VEPs, with high rates of visual conduction impairment in both subgroups (92.3% for PPMS and 85.7% for SPMS), significantly more elevated than the abnormalities recorded among the RRMS cohort (77.4%; Leocani et al., 2006). These findings were consistent with those deriving from other previous experiences: in a small Japanese cohort of 11 PPMS patients, higher frequencies of VEPs abnormality were reported in comparison with 35 RRMS patients (Kira et al., 1993). In a similar way, data extrapolated from a European cohort of 156 PPMS patients showed a delay of conduction along the visual pathway in 105 out of 131 subjects (80%) who had undergone ff-VEP examination (Stevenson et al., 1999); VEP studies in PMS patients are summarized in Table 1. The high frequency of abnormal ff-VEPs in PPMS, asymptomatic in the vast majority of the cases, allowed to reveal a clinically unsuspected spatial dissemination of the disease, and ff-VEPs were therefore once included among PPMS diagnostic criteria (Thompson et al., 2000). Multifocal VEP is a new technique that provides high sensitivity and specificity in detecting abnormalities in visual function in MS patients (Laron et al., 2009); however, no specific information exploring their role in PMS is currently available in literature to the knowledge of the authors.

**TABLE 1:** Studies assessing visual evoked potentials (VEPs) in progressive multiple sclerosis (PMS).

Study	Technique	Cohort	Main findings
Leocani et al., 2006	ff-VEPs	43 RRMS, 28 SPMS, 13 PPMS	VEPs abnormalities significantly more frequent in PMS (92.3% PPMS and 85.7% SPMS) than in RRMS (77.4%)
Kira et al., 1993	ff-VEPs	35 RRMS, 11 PPMS (japanese)	VEPs abnormalities more frequent in PPMS compared to RRMS patients
Stevenson et al., 1999	ff-VEPs	131 PPMS	Visual conduction delay in 105/131 (80%) PPMS patients

*ff-VEPs, full-field visual evoked potentials; RRMS, relapsing–remitting multiple sclerosis; SPMS, secondary progressive multiple sclerosis; PPMS, primary progressive multiple sclerosis.*

Backner et al. (2019) analyzed the relations between different vision-related measures, including ff-VEPs, in PMS. In particular, they reported data related to a cohort of 48 PMS patients (classified as 18 progressive with relapses, 21 SPMS, and nine PPMS) who had been enrolled in a longitudinal mesenchymal stem cell therapy study (NCT02166021), conducted at the Hadassah-Hebrew University Medical Center. Significant inverse correlations were found between motion perception tests [object for motion (OFM) and number for motion (NFM)] and ff-VEPs latency in eyes with previous ON and their fellow eyes, in the presence of preserved visual acuity (VA), thus confirming previous evidences suggesting that dynamic visual functions may reflect myelination levels along the visual pathway (Raz et al., 2014). Considering instead functional and structural measures, a correlation between ff-VEPs latency on the one hand and RNFL thickness as well as optic radiation lesion load on the other was described in non-ON eyes of the same cohort of patients enrolled in the NCT02166021 trial (Berman et al., 2020). In this regard, Davies et al. (1998) had previously reported optic nerve lesion length and area [detected by MRI on the short tau inversion recovery (STIR) sequence], to significantly correlate with ff-VEP latency prolongation in a cohort of 25 SPMS patients, only four of whom had a history of acute ON.

When considering the specific prognostic role of VEPs in PMS, available data are even more limited. Sater et al. (1999) proposed ff-VEPs as a tool to assess disease progression in addition to standard disability-based endpoints: obtaining serial VEPs and MRI scans from 11 PMS patients over a 1.5-year period, they found in fact no significant change in disability as measured by EDSS and Ambulation Index, nor in MRI T2 plaque burden, in the presence, however, of a significant progression of the P100 latency over time (Sater et al., 1999). More recently, Schlaeger et al. (2014) prospectively investigated the role of VEPs in the context of a multimodal evoked potential assessment as possible predictors of disease course in a small cohort of PPMS patients; they found that a multimodal evoked potential score correlated with disability in these patients, also allowing some prediction of the course of the disease.

## Optical Coherence Tomography in PMS

Several studies over the last 15 years examined cross-sectionally the pattern of retinal axonal loss (expressed by RNFL measurement at a peripapillary level), across the different MS clinical subtypes also including subsets of PMS patients, often coming to partially contrasting conclusions. As a premise, it is important to notice that early studies measured RNFL thickness through time-domain OCT devices (TD-OCT), while more recent experiences have been made with next-generation OCT based on spectral-domain technology (SD-OCT). This innovation allowed not only to increase speed acquisition but also to improve resolution power and reproducibility; segmentation algorithms also differ between TD-OCT and SD-OCT devices; therefore, results obtained with different OCT generations are not interchangeable and directly comparable (Bock et al., 2010).

In 2007, Pulicken et al. (2007) obtained RNFL thickness measures using a TD-OCT device (OCT-3, Zeiss Meditec) on a cohort of 135 RRMS, 16 SPMS, and 12 PPMS patients, as well as in 47 healthy controls: the three subgroups of MS patients all showed decreased RNFL values in comparison with controls; compared with RRMS, both SPMS and PPMS patients revealed a trend toward thinner RNFL values although in the absence of a statistical significance, probably due to the small number of PMS patients included in the study. In 2008, Henderson et al. (2008) performed a similar study (using TD-OCT Stratus, Zeiss Meditec) on 27 SPMS and 23 PPMS patients, with the former but not the latter showing reduced RNFL thickness values when compared with 20 healthy controls, in the absence of significant differences between the two PMS subgroups when age-adjusted regression coefficient of RNFL thickness was directly compared (although in the presence of lower values among SPMS patients); significant correlations between RNFL values and VA measures were also reported, especially in the PPMS cohort. In another study using the same TD-OCT device (Stratus) published in 2010, Siepman et al. (2010) reported no statistically significant difference in terms of mean RNFL thickness comparing 26 RRMS and 29 PPMS patients. In 2012, Gelfand et al. (2012) published retinal imaging data obtained in 60 SPMS and 33 PPMS patients, using a new SD-OCT device (Spectralis, Heidelberg Engineering): the authors reported similar RNFL thickness values between SPMS and PPMS patients examining eyes without previous ON, with TMV slightly lower in the PPMS group. These results were consistent with those published by Albrecht et al. (2012), including 41 SPMS and 12 PPMS patients: using the same Spectralis device, the authors reported significant RNFL thinning compared with healthy controls for both subgroups, although a direct comparison between different PMS subsets was not performed. Another coeval work performed with Spectralis on a German cohort of 414 MS patients (308 RRMS, 65 SPMS, and 41 PPMS) and 94 healthy controls reported significant differences in terms of RNFL thickness only between RRMS and SPMS patients after adjusting for clinical–demographic parameters (such as age, gender, and disease duration), while the PPMS subgroup did not differ from neither RRMS nor SPMS cohorts; a different pattern was obtained for TMV measures, for which a significant reduction was found in both SPMS and PPMS subgroups when compared with RRMS patients (Oberwahrenbrock et al., 2012). Data deriving from a Dutch cohort of 230 MS patients (including 61 SPMS and 29 PPMS), despite being obtained with the same SD-OCT (Spectralis), depicted another different situation: the authors found SPMS to show significant RNFL thickness reduction in comparison with PPMS but not RRMS patients, with PPMS subgroup showing the highest absolute values (Balk et al., 2014). Finally, in a recently published work, Jankowska-Lech et al. (2019; using SD-OCT Topcon OCT 1000 Mark II, Topcon) compared 26 RRMS with 22 PMS patients, finding significantly decreased RNFL thickness values in the latter subgroup only when taking into account also eyes with previous ON. The contrasting results emerging from the studies listed above may be partly related to the different techniques employed, with new SD-OCT showing higher resolution power, image quality, and reproducibility than the older TD-OCT devices (Bock et al., 2010); however, due to the relatively small sample sizes provided across the different studies, RNFL inter-individual variability among general and MS population, as well as the possibility of primary retinal pathology in a subset of MS patients, may have also played a role (Kallenbach and Frederiksen, 2007; Petzold et al., 2010; Serbecic et al., 2010; Saidha et al., 2011).

In more recent years, thanks to the availability of new commercial software allowing retinal automated segmentation, increasing attention has been directed toward the analysis of other retinal strata (particularly GCIPL) measured on macular scans; initial specific information is becoming available also for PMS cohorts. Some of the studies previously described already took into account these aspects: Albrecht et al. (2012) performed a manual segmentation of macular scans, reporting reduced GCIPL values in both SPMS and PPMS patients compared with controls; in PPMS subgroup, a significant reduction of the INL was also reported but not confirmed after the exclusion from the analysis of eyes with previous ON. Balk et al. (2014), using instead an automated software program, showed GCIPL to be significantly reduced among PPMS patients when compared with SPMS, also in the absence of previous ON history. Another work published in 2017 using SD-OCT (Cirrus 5000, Zeiss Meditec) compared 29 PMS with 84 RRMS patients, showing in the former subgroup significantly reduced thickness values not only for GCIPL but also when considering outer plexiform layer (OPL); included patients, however, were of non-Caucasian origin (Behbehani et al., 2017). Cross-sectional OCT studies assessing retinal layers in PMS are summarized in Table 2.

**TABLE 2:** Cross-sectional OCT studies assessing retinal layers in PMS.

Study	Device	Cohort	Main findings
Pulicken et al., 2007	TD-OCT (OCT-3, Zeiss Meditec)	1 3 5 R RMS, 16 SPMS, 12 PPMS, 47 HC	RNFLt reduced in MS groups compared to HC; statistical trend indicating thinner RNFLt in SPMS and PPMS compared to RRMS
Henderson et al., 2008	TD-OCT (Stratus, Zeiss Meditec)	2 7 SPMS, 23 PPMS, 20 HC	Mean RNFLt reduced in SPMS (but not PPMS) compared to HC
Siepman et al., 2010	TD-OCT (Stratus, Zeiss Meditec)	26 RRMS, 10 SPMS, 29 PPMS	Mean RNFLt no statistically different between RRMS and PPMS patients
Gelfand et al., 2012	SD-OCT (Spectralis, Heidelberg Engineering)	4 5 CIS, 403 RRMS, 60 SPMS, 33 PPMS, 53 HC	Mean RNFLt similar in SPMS and PPMS patients in non-ON eyes; TMV slighlty lower in PPMS group
Albrecht et al., 2012	SD-OCT (Spectralis, Heidelberg Engineering)	4 2 RRMS, 41 SPMS, 12 PPMS, 95 HC	Mean RNFLt and GCIPLt reduction in both SPMS and PPMS compared to HC; INLt reduction only in PPMS in comparison to HC
Oberwahrenbrock et al., 2012	SD-OCT (Spectralis, Heidelberg Engineering)	308 RRMS, 65 SPMS, 41 PPMS, 94 HC	Mean RNFLt lower in SPMS (but not PPMS) compared to RRMS; TMV reduced in both SPMS and PPMS compared to RRMS
Balk et al., 2014	SD-OCT (Spectralis, Heidelberg Engineering)	140 RRMS, 61 SPMS, 29 PPMS, 63 HC	Mean RNFLt, GCIPLt and INLt reduction in SPMS compared with PPMS but not RRMS considering non-ON eyes; highest absolute values in PPMS
Behbehani et al., 2017	SD-OCT (Cirrus 5000, Zeiss Meditec)	84 RRMS, 29 PMS, 38 HC (non-caucasian)	Mean RNFLt, GCIPLt and OPLt reduced in PMS compared to RRMS patients
Jankowska-Lech et al., 2019	SD-OCT (OCT 1000 Mark II, Topcon)	26 RRMS, 22 PMS, 31 HC	Mean RNFLt reduced in PMS compared to RRMS patients only when taking into account ON eyes

*TD-OCT, time domain–optical coherence tomography; SD-OCT, spectral domain–optical coherence tomography; CIS, clinically isolated syndrome; RRMS, relapsing–remitting multiple sclerosis; SPMS, secondary progressive multiple sclerosis; PPMS, primary progressive multiple sclerosis; HC, healthy controls; RNFLt, retinal nerve fiber layer thickness; TMV, total macular volume; GCIPLt, ganglion cells–inner plexiform layer thickness; INLt, inner nuclear layer thickness; OPLt, outer plexiform layer thickness; ON, optic neuritis.*

Researchers also focused on exploring the relation between retinal measures and clinical parameters; available data, however, are often non-specific for PMS, with major contributions (relative to visual function and global disability measures) deriving from some of the studies previously reported. Henderson et al. (2008) found a relationship between VA (both high- and low-contrast tests) and RNFL thickness in their PMS cohort, with particularly robust data in PPMS patients, as also suggested by the observation (with SD-OCT Spectralis) of a significant relation between low-contrast VA and GCIPL thickness in another cohort of 25 PPMS patients (Poretto et al., 2017). The same authors, however, did not identify any significant relation between RNFL thickness and disease duration, duration of the progressive phase, nor with EDSS (Henderson et al., 2008). A lack of a correlation between RNFL thickness and EDSS has been also reported with SD-OCT Spectralis in a cohort of 28 non-Caucasian SPMS patients (Yousefipour et al., 2016). Siepman et al. (2010) reported instead similar relations between RNFL thickness and VA, also pointing out a negative correlation with EDSS in eyes without previous ON; data, however, were referred to the entire study cohort of 26 RRMS and 29 PPMS patients. Albrecht et al. (2012) expanded this analysis in their cohort of 95 MS patients (including 41 SPMS and 12 PPMS) observing EDSS to correlate also with macular thickness and OPL, interestingly with a positive correlation in this latter case. Behbehani et al. (2017) reported instead an inverse correlation between ONL thickness and EDSS in 29 PMS patients. No significant correlation with RNFL (measured with Spectralis) was instead identified when considering motion perception tests, which appear to be mainly related to myelination status along the visual pathway more than to axonal loss (Backner et al., 2019). Finally, considering the possible relation between OCT measures and other clinical parameters, Coric et al. (2018) analyzed with Spectralis a cohort of 217 MS patients (including a remarkable percentage of PMS patients – 56 SPMS and 28 PPMS, respectively) describing cognitively impaired patients to have significantly reduced RNFL and GCIPL values.

Moving to assess the relation between OCT and other instrumental parameters, in 2007, Gordon-Lipkin et al. (2007) had already described RNFL thickness (measured with OCT-3) to correlate with brain atrophy in 40 MS patients (20 RRMS and 20 PMS), although this association appeared to be driven by the RRMS subset and by cerebrospinal fluid more than white or gray matter volume. In another cohort of 25 PPMS patients (assessed with Spectralis), RNFL thickness revealed to be associated with thalamus and visual cortex volume, while GCIPL values were associated with cortical lesion load; the authors suggested retrograde *trans*-synaptic degeneration and/or a common pathophysiologic process affecting both the brain and the retina as possible explanations (Petracca et al., 2017). Data deriving from a recent Italian retrospective study including a cohort of 84 PMS patients also revealed increased values of INL thickness in a subset of patients who had shown MRI activity during the year before OCT assessment (Spectralis), proposing INL evaluation as a possible surrogate marker of disease activity also among progressive patients (Cellerino et al., 2019). Saidha et al. (2015) explored the relation between SD-OCT (Cirrus 4000, Zeiss Meditec) and MRI parameters longitudinally in the context of a 4-year study including 107 MS patients: the authors described RNFL and GCIPL thinning to be significantly associated with whole-brain, and gray and white matter atrophy, pointing out a stronger relation in the subset of 36 PMS patients. However, data extrapolated from a randomized placebo-controlled trial testing the possible role of lipoic acid in SPMS showed only modest correlations between RNFL and cortical gray matter atrophy in a subset of 51 patients with OCT (Cirrus 5000) and MRI longitudinal data available, with no significant results for GCIPL (Winges et al., 2019). In the SPRINT MS phase II clinical trial, however, ibudilast significantly reduced over 2 years the progression of brain atrophy compared with placebo in PMS patients; this positive result was supported by a trend for a lower RNFL thickness reduction in ibudilast-treated patients (Fox et al., 2018). Finally, OCT parameters have been also analyzed in association with other functional instrumental techniques: in particular, a correlation between RNFL thickness and ff-VEPs latency has been identified in PMS patients considering eyes without ON history (Backner et al., 2019).

The evolution over time of OCT parameters has also started to be explored in different subsets of MS patients, but conclusive specific data for PMS are still lacking. In a work published in 2010, Talman et al. (2010) followed up (mean 18 months, range 6 months–4.5 years) 299 MS patients (84% with RRMS phenotype) with TD-OCT (OCT-3): the authors described progressive RNFL thinning as a function of time. In contrast with this finding, Henderson et al. (2010) using Stratus TD-OCT did not find any significant change of RNFL thickness over time in a small cohort of 34 PMS patients (18 SPMS and 16 PPMS) who were followed up for a median interval of 1.5 years. Balk et al. (2016) performed another study enrolling 135 MS patients (including 26 SPMS and 13 PPMS), who have been assessed with SD-OCT (Spectralis) over a 2-year period: the authors showed RNFL and GCIPL thinning to be significantly related to disease duration (with thinning rate becoming smaller in the presence of longer disease duration), and consistently, they found RNFL and GCIPL atrophy rate to be higher in RRMS than SPMS patients; such a relation was not identified for INL. Longitudinal data over 2 years relative to the cohort of 51 SPMS patients enrolled in the lipoic acid trial showed annualized RNFL and GCIPL atrophy rates (−0.31 and −0.29 μm/year, respectively) to not differ between eyes with and without previous ON history; however, a baseline RNFL thickness higher than 75 μm was associated with a greater (−0.85 μm/year) annualized atrophy rate (Winges et al., 2019). Only very recently Sotirchos et al. (2020) published a significant OCT longitudinal study including a cohort of 178 RRMS and 186 PMS patients who were followed up with serial OCT scans (performed with Cirrus SD device) for a median of 3.7 years: independently from age, PMS phenotype was found to be associated with faster mean annualized percent changes for both RNFL (−0.34%/year) and GCIPL (−0.27%/year), and possibly also for INL and ONL, with no significant impact determined by disease-modifying therapies; the relation between retinal layers atrophy rates and disability progression over time, however, has not been extensively assessed. Longitudinal OCT studies assessing evolution over time of retinal layers in PMS are summarized in Table 3.

**TABLE 3:** Longitudinal OCT studies assessing retinal layers in PMS.

Study	Device	Cohort	Follow-up	Main findings
Talman et al., 2010	TD-OCT (OCT-3, Zeiss Meditec)	299 MS (84% RRMS)	1.5 years (range 0.5–4.5)	RNFLt reduction as a function of time (average −2.9 μm at 2–3 years and −6.1 μm at 3–4.5 years) in some patients with MS, even in the absence of aON
Henderson et al., 2010	TD-OCT (Stratus, Zeiss Meditec)	18 SPMS, 16 PPMS, 18 HC	1.5 years (range 1.1–2.4)	No significant RNFLt reduction over time in patients and controls. TMV decline in both groups, with no between-group differences
Balk et al., 2016	SD-OCT (Spectralis, Heidelberg Engineering)	7 CIS, 89 RRMS, 26 SPMS, 13 PPMS, 16 HC	2 years	RNFLt and GCIPLt reductions more pronounced early in the course of disease (higher atrophy rate in RRMS than SPMS patients)
Winges et al., 2019	SD-OCT (Cirrus 5000, Zeiss Meditec)	51 SPMS	2 years	RNFL (−0.31 μm/year) and GCIPL (−0.29 μm/year) atrophy rates similar in aON and nON eyes; RNFLt > 75 μm associated with higher (−0.85 μm/year) rate
Sotirchos et al., 2020	SD-OCT (Cirrus HD-OCT, Zeiss Meditec)	178 RRMS, 186 PMS, 66 HC	3.7 years (IQ range 2.0–5.9)	PMS phenotype associated with faster RNFLt (−0.34 %/year) and GCIPLt (−0.27 %/year) reduction; no significant impact determined by DMTs

*TD-OCT, time domain–optical coherence tomography; SD-OCT, spectral domain–optical coherence tomography; CIS, clinically isolated syndrome; RRMS, relapsing–remitting multiple sclerosis; SPMS, secondary progressive multiple sclerosis; PPMS, primary progressive multiple sclerosis; HC, healthy controls; RNFLt, retinal nerve fiber layer thickness; TMV, total macular volume; GCIPLt, ganglion cells–inner plexiform layer thickness; aON, acute optic neuritis; nON, non-optic neuritis; DMTs, disease-modifying treatments.*

## Concluding Remarks

Optic pathway offers the unique opportunity to combine functional and structural measures: given the demonstrated correlations between optic nerve and brain damage (as revealed by MRI), it represents an attractive CNS area of interest to monitor MS evolution, as well as the response to DMTs, particularly in PMS. On the one hand, VEP studies, albeit in the presence of limited specific information, suggested a significant functional involvement of the visual pathway in PMS, in the presence of a relation with dynamic visual function measures and with a possible prognostic contribution on progression, in the context of a multimodal assessment of evoked responses. On the other hand, OCT studies, although in the presence of some contrasting results, highlighted a significant retinal neuro-axonal loss in PMS compared with HC but also RRMS patients, in the presence of possible, although non-linear, cross-sectional and longitudinal relations with measures of visual and global disability. Significant relations have been also identified in PMS between retinal neuro-axonal architecture and structural measures of brain atrophy provided by MRI; more recently, INL has been proposed as a marker of neuroinflammation also in the progressive phase of the disease. Our exploration of the literature, however, appears to highlight a lack of studies specifically combining a functional exploration of the visual pathway with a morphological description of the retina in PMS patients, with a still open possibility to better characterize the relation between demyelination and neurodegeneration in the progressive phase of the disease. To validate the use of VEPs and OCT in PMS, it is mandatory to recruit large cohorts of patients in the context of multicenter studies, longitudinally followed to define the correlations with clinically relevant visual parameters from the one side (i.e., contrast sensitivity measures) and with global disability measures on the other. Of great value could be also studies comparing combined OCT and VEPs data with conventional and advanced MRI techniques. A better knowledge in the field would be of fundamental importance in a near future, in order to identify the most suitable biomarkers to assess the efficacy of possible neuroprotective and remyelinating strategies aimed to contrast irreversible disability accrual affecting PMS patients.

## Author Contributions

All listed authors have made a substantial, direct and intellectual contribution to the present work, and approved it for publication. In particular, SG performed literature search and wrote the first draft of the manuscript. LL and GC completed bibliography and revised the manuscript.

## Conflict of Interest

The authors declare that the research was conducted in the absence of any commercial or financial relationships that could be construed as a potential conflict of interest.

## Publisher’s Note

All claims expressed in this article are solely those of the authors and do not necessarily represent those of their affiliated organizations, or those of the publisher, the editors and the reviewers. Any product that may be evaluated in this article, or claim that may be made by its manufacturer, is not guaranteed or endorsed by the publisher.

## Footnotes

1
www.progressivemsalliance.org

## References

[B1] Albrecht P. Frohlich R. Hartung H. P. Kieseier B. C. Methner A. (2007). Optical coherence tomography measures axonal loss in multiple sclerosis independently of optic neuritis. *J. Neurol.* 254 1595–1596. 10.1007/s00415-007-0538-3 17987252

[B2] Albrecht P. Ringelstein M. Muller A. K. Keser N. Dietlein T. Lappas A. (2012). Degeneration of retinal layers in multiple sclerosis subtypes quantified by optical coherence tomography. *Mult. Scler.* 18 1422–1429. 10.1177/1352458512439237 22389411

[B3] Alonso R. Gonzalez-Moron D. Garcea O. (2018). Optical coherence tomography as a biomarker of neurodegeneration in multiple sclerosis: a review. *Mult. Scler. Relat. Disord.* 22 77–82. 10.1016/j.msard.2018.03.007 29605802

[B4] Backner Y. Petrou P. Glick-Shames H. Raz N. Zimmermann H. Jost R. (2019). Vision and vision-related measures in progressive multiple sclerosis. *Front. Neurol.* 10:455. 10.3389/fneur.2019.00455 31130910 PMC6509148

[B5] Balk L. J. Cruz-Herranz A. Albrecht P. Arnow S. Gelfand J. M. Tewarie P. (2016). Timing of retinal neuronal and axonal loss in MS: a longitudinal OCT study. *J. Neurol.* 263 1323–1331. 10.1007/s00415-016-8127-y 27142714 PMC4929170

[B6] Balk L. J. Coric D. Knier B. Zimmermann H. G. Behbehani R. Alroughani R. (2019). Retinal inner nuclear layer volume reflects inflammatory disease activity in multiple sclerosis; a longitudinal OCT study. *Mult. Scler. J. Exp. Transl. Clin.* 5:2055217319871582. 10.1177/2055217319871582 31523449 PMC6728683

[B7] Balk L. J. Tewarie P. Killestein J. Polman C. H. Uitdehaag B. Petzold A. (2014). Disease course heterogeneity and OCT in multiple sclerosis. *Mult. Scler.* 20 1198–1206. 10.1177/1352458513518626 24402036

[B8] Behbehani R. Abu Al-Hassan A. Al-Salahat A. Sriraman D. Oakley J. D. Alroughani R. (2017). Optical coherence tomography segmentation analysis in relapsing remitting versus progressive multiple sclerosis. *PLoS One* 12:e0172120. 10.1371/journal.pone.0172120 28192539 PMC5305239

[B9] Berman S. Backner Y. Krupnik R. Paul F. Petrou P. Karussis D. (2020). Conduction delays in the visual pathways of progressive multiple sclerosis patients covary with brain structure. *Neuroimage* 221:117204. 10.1016/j.neuroimage.2020.117204 32745679

[B10] Bock M. Brandt A. U. Dörr J. Pfueller C. F. Ohlraun S. Zipp F. (2010). Time domain and spectral domain optical coherence tomography in multiple sclerosis: a comparative cross-sectional study. *Mult. Scler.* 16 893–896. 10.1177/1352458510365156 20350961

[B11] Bsteh G. Hegen H. Altmann P. Auer M. Berek K. Di Pauli F. (2020). Retinal layer thinning is reflecting disability progression independent of relapse activity in multiple sclerosis. *Mult. Scler. J. Exp. Transl. Clin.* 6:2055217320966344. 10.1177/2055217320966344 33194221 PMC7604994

[B12] Cellerino M. Cordano C. Boffa G. Bommarito G. Petracca M. Sbragia E. (2019). Relationship between retinal inner nuclear layer, age, and disease activity in progressive MS. *Neurol. Neuroimmunol. Neuroinflamm.* 6:e596. 10.1212/NXI.0000000000000596 31454778 PMC6705649

[B13] Chatziralli I. P. Moschos M. M. Brouzas D. Kopsidas K. Ladas I. D. (2012). Evaluation of retinal nerve fibre layer thickness and visual evoked potentials in optic neuritis associated with multiple sclerosis. *Clin. Exp. Optom.* 95 223–228. 10.1111/j.1444-0938.2012.00706.x 22329676

[B14] Chen L. Gordon L. K. (2005). Ocular manifestations of multiple sclerosis. *Curr. Opin. Ophthalmol.* 16 315–320. 10.1097/01.icu.0000179804.49842.e2 16175046

[B15] Comi G. Leocani L. Medaglini S. Locatelli T. Martinelli V. Santuccio G. (1999). Measuring evoked responses in multiple sclerosis. *Mult. Scler.* 5 263–267. 10.1177/135245859900500412 10467386

[B16] Confavreux C. Vukusic S. (2006). Natural history of multiple sclerosis: a unifying concept. *Brain* 129(Pt 3) 606–616. 10.1093/brain/awl007 16415308

[B17] Coric D. Balk L. J. Verrijp M. Eijlers A. Schoonheim M. M. Killestein J. (2018). Cognitive impairment in patients with multiple sclerosis is associated with atrophy of the inner retinal layers. *Mult. Scler.* 24 158–166. 10.1177/1352458517694090 28273785 PMC5987993

[B18] Correale J. Gaitan M. I. Ysrraelit M. C. Fiol M. P. (2017). Progressive multiple sclerosis: from pathogenic mechanisms to treatment. *Brain* 140 527–546. 10.1093/brain/aww258 27794524

[B19] Costello F. Burton J. M. (2018). Retinal imaging with optical coherence tomography: a biomarker in multiple sclerosis? *Eye Brain* 10 47–63. 10.2147/EB.S139417 30104912 PMC6074809

[B20] Davies M. B. Williams R. Haq N. Pelosi L. Hawkins C. P. (1998). MRI of optic nerve and postchiasmal visual pathways and visual evoked potentials in secondary progressive multiple sclerosis. *Neuroradiology* 40 765–770. 10.1007/s002340050681 9877128

[B21] Di Maggio G. Santangelo R. Guerrieri S. Bianco M. Ferrari L. Medaglini S. (2014). Optical coherence tomography and visual evoked potentials: which is more sensitive in multiple sclerosis? *Mult. Scler.* 20 1342–1347. 10.1177/1352458514524293 24591532

[B22] Diebold M. Derfuss T. (2016). Immunological treatment of multiple sclerosis. *Semin. Hematol.* 53(Suppl. 1) S54–S57. 10.1053/j.seminhematol.2016.04.016 27312167

[B23] Fisher J. B. Jacobs S. L. Markowitz D. A. Galetta C. E. Volpe N. J. Nano-Schiavi M. L. (2006). Relation of visual function to retinal nerve fiber layer thickness in multiple sclerosis. *Ophthalmology* 113 324–332. 10.1016/j.ophtha.2005.10.040 16406539

[B24] Fox R. J. Coffey C. S. Conwit R. Cudkowicz M. E. Gleason T. Goodman A. (2018). Phase 2 trial of ibudilast in progressive multiple sclerosis. *N. Engl. J. Med.* 379 846–855. 10.1056/NEJMoa1803583 30157388 PMC6172944

[B25] Frischer J. M. Bramow S. Dal-Bianco A. Lucchinetti C. F. Rauschka H. Schmidbauer M. (2009). The relation between inflammation and neurodegeneration in multiple sclerosis brains. *Brain* 132(Pt 5) 1175–1189. 10.1093/brain/awp070 19339255 PMC2677799

[B26] Gabilondo I. E. Martinez-Lapiscina H. Martinez-Heras E. Fraga-Pumar E. Llufriu S. Ortiz S. (2014). Trans-synaptic axonal degeneration in the visual pathway in multiple sclerosis. *Ann. Neurol.* 75 98–107. 10.1002/ana.24030 24114885

[B27] Gelfand J. M. Goodin D. S. Boscardin W. J. Nolan R. Cuneo A. Green A. J. (2012). Retinal axonal loss begins early in the course of multiple sclerosis and is similar between progressive phenotypes. *PLoS One* 7:e36847. 10.1371/journal.pone.0036847 22666330 PMC3359324

[B28] Giovannoni G. Butzkueven H. Dhib-Jalbut S. Hobart J. Kobelt G. Pepper G. (2016). Brain health: time matters in multiple sclerosis. *Mult. Scler. Relat. Disord.* 9(Suppl. 1) S5–S48. 10.1016/j.msard.2016.07.003 27640924

[B29] Gordon-Lipkin E. Chodkowski B. Reich D. S. Smith S. A. Pulicken M. Balcer L. J. (2007). Retinal nerve fiber layer is associated with brain atrophy in multiple sclerosis. *Neurology* 69 1603–1609. 10.1212/01.wnl.0000295995.46586.ae 17938370

[B30] Grazioli E. Zivadinov R. Weinstock-Guttman B. Lincoff N. Baier M. Wong J. R. (2008). Retinal nerve fiber layer thickness is associated with brain MRI outcomes in multiple sclerosis. *J. Neurol. Sci.* 268 12–17. 10.1016/j.jns.2007.10.020 18054962

[B31] Green A. J. McQuaid S. Hauser S. L. Allen I. V. Lyness R. (2010). Ocular pathology in multiple sclerosis: retinal atrophy and inflammation irrespective of disease duration. *Brain* 133(Pt 6) 1591–1601. 10.1093/brain/awq080 20410146 PMC2877904

[B32] Heesen C. Bohm J. Reich C. Kasper J. Goebel M. Gold S. M. (2008). Patient perception of bodily functions in multiple sclerosis: gait and visual function are the most valuable. *Mult. Scler.* 14 988–991. 10.1177/1352458508088916 18505775

[B33] Henderson A. P. Trip S. A. Schlottmann P. G. Altmann D. R. Garway-Heath D. F. Plant G. T. (2008). An investigation of the retinal nerve fibre layer in progressive multiple sclerosis using optical coherence tomography. *Brain* 131(Pt 1) 277–287. 10.1093/brain/awm285 18056739

[B34] Henderson A. P. Trip S. A. Schlottmann P. G. Altmann D. R. Garway-Heath D. F. Plant G. T. (2010). A preliminary longitudinal study of the retinal nerve fiber layer in progressive multiple sclerosis. *J. Neurol.* 257 1083–1091. 10.1007/s00415-010-5467-x 20143110

[B35] International Progressive MS Alliance (2021). *What We Do.* Available online at: https://www.progressivemsalliance.org/what-we-do (accessed March 22, 2021).

[B36] Jankowska-Lech I. Wasyluk J. Palasik W. Terelak-Borys B. Grabska-Liberek I. (2019). Peripapillary retinal nerve fiber layer thickness measured by optical coherence tomography in different clinical subtypes of multiple sclerosis. *Mult. Scler. Relat. Disord.* 27 260–268. 10.1016/j.msard.2018.11.003 30423530

[B37] Kallenbach K. Frederiksen J. (2007). Optical coherence tomography in optic neuritis and multiple sclerosis: a review. *Eur. J. Neurol.* 14 841–849. 10.1111/j.1468-1331.2007.01736.x 17662003

[B38] Kappos L. Bar-Or A. Cree B. A. C. Fox R. J. Giovannoni G. Gold R. (2018). Siponimod versus placebo in secondary progressive multiple sclerosis (EXPAND): a double-blind, randomised, phase 3 study. *Lancet* 391 1263–1273. 10.1016/S0140-6736(18)30475-6 29576505

[B39] Kaushik M. Yu Wang C. Barnett M. H. Garrick R. Parratt J. Graham S. L. (2013). Inner nuclear layer thickening is inversley proportional to retinal ganglion cell loss in optic neuritis. *PLoS One* 8:e78341. 10.1371/journal.pone.0078341 24098599 PMC3789678

[B40] Kerrison J. B. Flynn T. Green W. R. (1994). Retinal pathologic changes in multiple sclerosis. *Retina* 14 445–451. 10.1097/00006982-199414050-00010 7899721

[B41] Kira J. Tobimatsu S. Goto I. Hasuo K. (1993). Primary progressive versus relapsing remitting multiple sclerosis in Japanese patients: a combined clinical, magnetic resonance imaging and multimodality evoked potential study. *J. Neurol. Sci.* 117 179–185. 10.1016/0022-510x(93)90171-t 8410054

[B42] Klistorner A. Arvind H. Nguyen T. Garrick R. Paine M. Graham S. (2008). Axonal loss and myelin in early ON loss in postacute optic neuritis. *Ann. Neurol.* 64 325–331. 10.1002/ana.21474 18825673

[B43] Knier B. Schmidt P. Aly L. Buck D. Berthele A. Mühlau M. (2016). Retinal inner nuclear layer volume reflects response to immunotherapy in multiple sclerosis. *Brain* 139 2855–2863. 10.1093/brain/aww219 27581073

[B44] Kolbe S. C. Marriott M. Walt A. Fielding J. Klistorner A. Mitchell P. J. (2012). Diffusion tensor imaging correlates of visual impairment in multiple sclerosis and chronic optic neuritis. *Invest. Ophthalmol. Vis. Sci.* 53 825–832. 10.1167/iovs.11-8864 22247457

[B45] Lambe J. Fitzgerald K. C. Murphy O. C. Filippatou A. G. Sotirchos E. S. Kalaitzidis G. (2021). Association of spectral-domain OCT with long-term disability worsening in multiple sclerosis. *Neurology* 96 e2058–e2069. 10.1212/WNL.0000000000011788 33653904 PMC8166450

[B46] Laron M. Cheng H. Zhang B. Schiffman J. S. Tang R. A. Frishman L. J. (2009). Assessing visual pathway function in multiple sclerosis patients with multifocal visual evoked potentials. *Mult. Scler.* 15 1431–1441. 10.1177/1352458509350470 19995841 PMC2933374

[B47] Lassmann H. (2017). Targets of therapy in progressive MS. *Mult. Scler.* 23 1593–1599. 10.1177/1352458517729455 29041864

[B48] Lassmann H. Bruck W. Lucchinetti C. F. (2007). The immunopathology of multiple sclerosis: an overview. *Brain Pathol.* 17 210–218. 10.1111/j.1750-3639.2007.00064.x 17388952 PMC8095582

[B49] Lassmann H. van Horssen J. Mahad D. (2012). Progressive multiple sclerosis: pathology and pathogenesis. *Nat. Rev. Neurol.* 8 647–656. 10.1038/nrneurol.2012.168 23007702

[B50] Leocani L. Guerrieri S. Comi G. (2018). Visual evoked potentials as a biomarker in multiple sclerosis and associated optic neuritis. *J. Neuroophthalmol.* 38 350–357. 10.1097/WNO.0000000000000704 30106802

[B51] Leocani L. Rovaris M. Boneschi F. M. Medaglini S. Rossi P. Martinelli V. (2006). Multimodal evoked potentials to assess the evolution of multiple sclerosis: a longitudinal study. *J. Neurol. Neurosurg. Psychiatry* 77 1030–1035. 10.1136/jnnp.2005.086280 16735397 PMC2077734

[B52] Lublin F. D. Reingold S. C. (1996). Defining the clinical course of multiple sclerosis: results of an international survey. National Multiple Sclerosis Society (USA) Advisory Committee on clinical trials of new agents in multiple sclerosis. *Neurology* 46 907–911. 10.1212/wnl.46.4.907 8780061

[B53] Lublin F. D. Reingold S. C. Cohen J. A. Cutter G. R. Sørensen P. S. Thompson A. J. (2014). Defining the clinical course of multiple sclerosis: the 2013 revisions. *Neurology* 83 278–286. 10.1212/WNL.0000000000000560 24871874 PMC4117366

[B54] Martinez-Lapiscina E. H. Arnow S. Wilson J. A. Saidha S. Preiningerova J. L. Oberwahrenbrock T. (2016). Retinal thickness measured with optical coherence tomography and risk of disability worsening in multiple sclerosis: a cohort study. *Lancet Neurol.* 15 574–584. 10.1016/S1474-4422(16)00068-5 27011339

[B55] Martinez-Lapiscina E. H. Sanchez-Dalmau B. Fraga-Pumar E. Ortiz-Perez S. Tercero-Uribe A. I. Torres-Torres R. (2014). The visual pathway as a model to understand brain damage in multiple sclerosis. *Mult. Scler.* 20 1678–1685. 10.1177/1352458514542862 25013155

[B56] Melzi L. Rocca M. A. Bianchi-Marzoli S. Falini A. Vezzulli P. Ghezzi A. (2007). A longitudinal conventional and magnetization transfer magnetic resonance imaging study of optic neuritis. *Mult. Scler.* 13 265–268. 10.1177/1352458506071212 17439896

[B57] Montalban X. Hauser S. L. Kappos L. Arnold D. L. Bar-Or A. Comi G. (2017). Ocrelizumab versus placebo in primary progressive multiple sclerosis. *N. Engl. J. Med.* 376 209–220. 10.1056/NEJMoa1606468 28002688

[B58] Montolío A. Cegoñino J. Orduna E. Sebastian B. Garcia-Martin E. Pérez Del Palomar A. (2019). A mathematical model to predict the evolution of retinal nerve fiber layer thinning in multiple sclerosis patients. *Comput. Biol. Med.* 111:103357. 10.1016/j.compbiomed.2019.103357 31326867

[B59] Naismith R. T. Xu J. Tutlam N. T. Scully P. T. Trinkaus K. Snyder A. Z. (2010). Increased diffusivity in acute multiple sclerosis lesions predicts risk of black hole. *Neurology* 74 1694–1701. 10.1212/WNL.0b013e3181e042c4 20498437 PMC2882210

[B60] Oberwahrenbrock T. Schippling S. Ringelstein M. Kaufhold F. Zimmermann H. Keser N. (2012). Retinal damage in multiple sclerosis disease subtypes measured by high-resolution optical coherence tomography. *Mult. Scler. Int.* 2012:530305. 10.1155/2012/530305 22888431 PMC3410317

[B61] Ontaneda D. Fox R. J. (2015). Progressive multiple sclerosis. *Curr. Opin. Neurol.* 28 237–243. 10.1097/WCO.0000000000000195 25887766 PMC4425257

[B62] Ontaneda D. Fox R. J. Chataway J. (2015). Clinical trials in progressive multiple sclerosis: lessons learned and future perspectives. *Lancet Neurol.* 14 208–223. 10.1016/S1474-4422(14)70264-9 25772899 PMC4361791

[B63] Petracca M. Cordano C. Cellerino M. Button J. Krieger S. Vancea R. (2017). Retinal degeneration in primary-progressive multiple sclerosis: a role for cortical lesions? *Mult. Scler.* 23 43–50. 10.1177/1352458516637679 26993116

[B64] Petzold A. de Boer J. F. Schippling S. Vermersch P. Kardon R. Green A. (2010). Optical coherence tomography in multiple sclerosis: a systematic review and meta-analysis. *Lancet Neurol.* 9 921–932. 10.1016/S1474-4422(10)70168-X 20723847

[B65] Pisa M. Guerrieri S. Di Maggio G. Medaglini S. Moiola L. Martinelli V. (2017). No evidence of disease activity is associated with reduced rate of axonal retinal atrophy in MS. *Neurology* 89 2469–2475. 10.1212/WNL.0000000000004736 29142087

[B66] Pisa M. Ratti F. Vabanesi M. Radaelli M. Guerrieri S. Moiola L. (2020). Subclinical neurodegeneration in multiple sclerosis and neuromyelitis optica spectrum disorder revealed by optical coherence tomography. *Mult. Scler.* 26 1197–1206. 10.1177/1352458519861603 31392924

[B67] Poretto V. Petracca M. Saiote C. Mormina E. Howard J. Miller A. (2017). A composite measure to explore visual disability in primary progressive multiple sclerosis. *Mult. Scler. J. Exp. Transl. Clin.* 3:2055217317709620. 10.1177/2055217317709620 PMC5439656 28607759

[B68] Pueyo V. Martin J. Fernandez J. Almarcegui C. Ara J. Egea C. (2008). Axonal loss in the retinal nerve fiber layer in patients with multiple sclerosis. *Mult. Scler.* 14 609–614. 10.1177/1352458507087326 18424482

[B69] Pulicken M. Gordon-Lipkin E. Balcer L. J. Frohman E. Cutter G. Calabresi P. A. (2007). Optical coherence tomography and disease subtype in multiple sclerosis. *Neurology* 69 2085–2092. 10.1212/01.wnl.0000294876.49861.dc 18040015

[B70] Raz N. Hallak M. Ben-Hur T. Levin N. (2014). Dynamic visual tests to identify and quantify visual damage and repair following demyelination in optic neuritis patients. *J. Vis. Exp.* 86:e51107. 10.3791/51107 24798084 PMC4168837

[B71] Rothman A. Murphy O. C. Fitzgerald K. C. Button J. Gordon-Lipkin E. Ratchford J. N. (2019). Retinal measurements predict 10-year disability in multiple sclerosis. *Ann. Clin. Transl. Neurol.* 6 222–232. 10.1002/acn3.674 30847355 PMC6389740

[B72] Saidha S. Al-Louzi O. Ratchford J. N. Bhargava P. Oh J. Newsome S. D. (2015). Optical coherence tomography reflects brain atrophy in multiple sclerosis: a four-year study. *Ann. Neurol.* 78 801–813. 10.1002/ana.24487 26190464 PMC4703093

[B73] Saidha S. Sotirchos E. S. Ibrahim M. A. Crainiceanu C. M. Gelfand J. M. Sepah Y. J. (2012). Microcystic macular oedema, thickness of the inner nuclear layer of the retina, and disease characteristics in multiple sclerosis: a retrospective study. *Lancet Neurol.* 11 963–972. 10.1016/S1474-4422(12)70213-2 23041237 PMC3533139

[B74] Saidha S. Syc S. B. Ibrahim M. A. Eckstein C. Warner C. V. Farrell S. K. (2011). Primary retinal pathology in multiple sclerosis as detected by optical coherence tomography. *Brain* 134(Pt 2) 518–533. 10.1093/brain/awq346 21252110

[B75] Sater R. A. Rostami A. M. Galetta S. Farber R. E. Bird S. J. (1999). Serial evoked potential studies and MRI imaging in chronic progressive multiple sclerosis. *J. Neurol. Sci.* 171 79–83. 10.1016/s0022-510x(99)00255-5 10581371

[B76] Schlaeger R. D’Souza M. Schindler C. Grize L. Kappos L. Fuhr P. (2014). Electrophysiological markers and predictors of the disease course in primary progressive multiple sclerosis. *Mult. Scler.* 20 51–56. 10.1177/1352458513490543 23756680

[B77] Seigo M. A. Sotirchos E. S. Newsome S. Babiarz A. Eckstein C. Ford E. (2012). In vivo assessment of retinal neuronal layers in multiple sclerosis with manual and automated optical coherence tomography segmentation techniques. *J. Neurol.* 259 2119–2130. 10.1007/s00415-012-6466-x 22418995

[B78] Serbecic N. Aboul-Enein F. Beutelspacher S. C. Graf M. Kircher K. Geitzenauer W. (2010). Heterogeneous pattern of retinal nerve fiber layer in multiple sclerosis. High resolution optical coherence tomography: potential and limitations. *PLoS One* 5:e13877. 10.1371/journal.pone.0013877 21079732 PMC2975633

[B79] Siepman T. A. Bettink-Remeijer M. W. Hintzen R. Q. (2010). Retinal nerve fiber layer thickness in subgroups of multiple sclerosis, measured by optical coherence tomography and scanning laser polarimetry. *J. Neurol.* 257 1654–1660. 10.1007/s00415-010-5589-1 20461397 PMC2951505

[B80] Siger M. Dziegielewski K. Jasek L. Bieniek M. Nicpan A. Nawrocki J. (2008). Optical coherence tomography in multiple sclerosis: thickness of the retinal nerve fiber layer as a potential measure of axonal loss and brain atrophy. *J. Neurol.* 255 1555–1560. 10.1007/s00415-008-0985-5 18825432

[B81] Sorensen P. S. Fox R. J. Comi G. (2020). The window of opportunity for treatment of progressive multiple sclerosis. *Curr. Opin. Neurol.* 33 262–270. 10.1097/WCO.0000000000000811 32251026

[B82] Sotirchos E. S. Gonzalez Caldito N. Filippatou A. Fitzgerald K. C. Murphy O. C. Lambe J. (2020). Progressive multiple sclerosis is associated with faster and specific retinal layer atrophy. *Ann. Neurol.* 87 885–896. 10.1002/ana.25738 32285484 PMC8682917

[B83] Stevenson V. L. Miller D. H. Rovaris M. Barkhof F. Brochet B. Dousset V. (1999). Primary and transitional progressive MS: a clinical and MRI cross-sectional study. *Neurology* 52 839–845. 10.1212/wnl.52.4.839 10078736

[B84] Syc S. B. Saidha S. Newsome S. D. Ratchford J. N. Levy M. Ford E. (2012). Optical coherence tomography segmentation reveals ganglion cell layer pathology after optic neuritis. *Brain* 135 521–533. 10.1093/brain/awr264 22006982 PMC3281477

[B85] Talman L. S. Bisker E. R. Sackel D. J. Long D. A. Jr. Galetta K. M. Ratchford J. N. (2010). Longitudinal study of vision and retinal nerve fiber layer thickness in multiple sclerosis. *Ann. Neurol.* 67 749–760. 10.1002/ana.22005 20517936 PMC2901775

[B86] Tavazzi E. Jakimovski D. Kuhle J. Hagemeier J. Ozel O. Ramanathan M. (2020). Serum neurofilament light chain and optical coherence tomography measures in MS: a longitudinal study. *Neurol. Neuroimmunol. Neuroinflamm.* 7:e737. 10.1212/NXI.0000000000000737 32424064 PMC7251512

[B87] Thompson A. J. Montalban X. Barkhof F. Brochet B. Filippi M. Miller D. H. (2000). Diagnostic criteria for primary progressive multiple sclerosis: a position paper. *Ann. Neurol.* 47 831–835. 10852554

[B88] Trapp B. D. Nave K. A. (2008). Multiple sclerosis: an immune or neurodegenerative disorder? *Annu. Rev. Neurosci.* 31 247–269. 10.1146/annurev.neuro.30.051606.094313 18558855

[B89] Trip S. A. Schlottmann P. G. Jones S. J. Li W. Y. Garway-Heath D. F. Thompson A. J. (2006). Optic nerve atrophy and retinal nerve fibre layer thinning following optic neuritis: evidence that axonal loss is a substrate of MRI-detected atrophy. *Neuroimage* 31 286–293. 10.1016/j.neuroimage.2005.11.051 16446103

[B90] Vabanesi M. Pisa M. Guerrieri S. Moiola L. Radaelli M. Medaglini S. (2019). In vivo structural and functional assessment of optic nerve damage in neuromyelitis optica spectrum disorders and multiple sclerosis. *Sci. Rep.* 9:10371. 10.1038/s41598-019-46251-3 31316082 PMC6637174

[B91] Villoslada P. (2016). Neuroprotective therapies for multiple sclerosis and other demyelinating diseases. *Mult. Scler. Demyelinating Disord.* 1:1. 10.1186/s40893-016-0004-0

[B92] Winges K. M. Murchison C. F. Bourdette D. N. Spain R. I. (2019). Longitudinal optical coherence tomography study of optic atrophy in secondary progressive multiple sclerosis: results from a clinical trial cohort. *Mult. Scler.* 25 55–62. 10.1177/1352458517739136 29111873 PMC5930161

[B93] Yousefipour G. Hashemzahi Z. Yasemi M. Jahani P. (2016). Findings of optical coherence tomography of retinal nerve fiber layer in two common types of multiple sclerosis. *Acta Med. Iran.* 54 382–390. 27306345

[B94] Zaveri M. S. Conger A. Salter A. Frohman T. C. Galetta S. L. Markowitz C. E. (2008). Retinal imaging by laser polarimetry and optical coherence tomography evidence of axonal degeneration in multiple sclerosis. *Arch. Neurol.* 65 924–928. 10.1001/archneur.65.7.924 18625859

